# Enhancing Bidirectional Sulfur Conversion Through p–d Orbital Hybridization via Vacancy Engineering

**DOI:** 10.1002/EXP.20240362

**Published:** 2025-08-26

**Authors:** Yan Chen, Dan Li, Yufang Chen, Xingqiao Wu, Manfang Chen, Yuchao Du, Keyang Fu, Hao Yuan, Shuangying Wei, Xianyou Wang, Hongbo Shu

**Affiliations:** ^1^ Hunan Province Key Laboratory for Electrochemical Energy Storage and Conversion National Base for International Science & Technology Cooperation National Local Joint Engineering Laboratory for Key Materials of New Energy Storage Battery Key Laboratory of Environmentally Friendly Chemistry and Applications of Ministry of Education School of Chemistry Xiangtan University Xiangtan China; ^2^ College of Aerospace Science and Engineering National University of Defense Technology Changsha China; ^3^ Institute for Carbon Neutralization Technology College of Chemistry and Materials Engineering Wenzhou University Wenzhou Zhejiang China; ^4^ ZTE Corporation Shenzhen P. R. China; ^5^ Department of Industrial Chemistry University of Bologna Bologna Italy

**Keywords:** bidirectional catalysis, lithium–sulfur batteries, oxygen vacancy

## Abstract

Lithium–sulfur batteries (LSBs) have garnered significant concern as materials with high energy density for energy storage. Nevertheless, their severe shuttle effect and delayed redox kinetics limit their practical application. Herein, a strategy based on the regulation of oxygen vacancy concentration in CoWO_4_ has been proposed to accelerate polysulfide kinetics. Experiments and density functional theory calculations reveal that catalytic materials with the appropriate number of oxygen vacancies (CWO‐M) have moderate adsorption energy and optimal catalytic capacity for polysulfides due to strong p–d orbital hybridization. More importantly, CWO‐M not only accelerates the reduction of sulfur during discharge but also significantly accelerates the oxidation of Li_2_S during charging, showing a favorable bidirectional catalytic effect. Benefiting from these unique advantages, the CWO‐M/S‐based battery exhibits an excellent rate performance of 768 mAh g^−1^ at 2 C and a capacity retention of 91.1% after 100 cycles at 0.2 C. Stable cycling performance with a high capacity of nearly 4 mAh cm^−2^ was achieved even after 100 cycles at a high sulfur loading of 8.02 mg cm^−2^ and a low electrolyte/sulfur (E/S) ratio of 8 µL mg S^−1^. This work provides significant insights into bidirectional catalysts by modulating the oxygen vacancy concentration for application in LSBs.

## Graphical abstract

To effectively alleviate the severe shuttle effect and delayed redox kinetics of lithium–sulfur batteries, CoWO_4−_ *_x_*/CNT composites with different oxygen vacancy concentrations were prepared. The p–d orbital hybridization caused by oxygen vacancy enhances the bidirectional sulfur conversion, and provides valuable insights for the development of highly active catalysts for lithium–sulfur batteries.

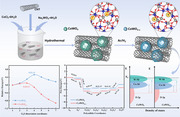

## 1 Introduction

The rapid and sustainable development of electric vehicles and consumer electronics has accelerated the development of novel battery systems with high energy density [1]. Among the systems, lithium–sulfur batteries (LSBs) have attracted significant interest owing to their high energy density (2600 Wh kg^−1^) and substantial theoretical specific capacity (1675 mAh g^−1^) [2, 3, 4]. Furthermore, sulfur resources are inexpensive, plentiful, and environmentally benign [5]. Based on these advantages, it is essential to develop LSBs to supplement energy storage systems. However, the poor sulfur utilization and dissatisfactory cycling stability of LSBs have hindered the broader adoption of LSBs due to the inadequate electrical conductivity of sulfur and its reduction products, as well as the slow reaction kinetics and severe shuttle effect of lithium polysulfide (LiPSs) [6, 7, 8].

The commonly reported methods of physical confinement [9] and chemical adsorption [10] are unable to address the aforementioned problems because of their low affinity for LiPSs and inferior ability to quicken up the conversion of sulfur species. The aforementioned obstacles can be solved by introducing electrocatalysts to optimize the electrochemical reaction kinetics of long‐chain sulfur species and decrease their presence or migration in the electrolyte [11]. Nonetheless, from the fundamental perspective, intricate LiPSs transformations and multistep electron‐transfer processes accompany the entire discharge and charge processes of LSBs [12, 13], that is, discharge: a solid (S_8_) → liquid (Li_2_S_*n*_) →solid (Li_2_S_2_/Li_2_S); charge: Li_2_S_2_/Li_2_S is first oxidized to LiPSs and eventually to S_8_. The electron and energy requirements for the sulfur reduction reaction (SRR) and sulfur evolution reaction (SER) are different [14]. Therefore, conventional single catalysts may not be able to maximize the acceleration of the bidirectional sulfur conversion reaction. Despite considerable efforts on simple combinations of different catalytic materials (e.g., construction of heterojunctions [15, 16, 17]) have been employed to overcome their respective drawbacks in stepwise SRR and SER, the improvements in reaction kinetics are still restricted by inadequate charge transfer and interfacial contacts between two or more substances. Consequently, the design of a single catalyst material that facilitates both SRR and SER processes is key to the solution.

Vacancies in engineering are regarded as an efficient tactic for surface modification of electrocatalysts. Defective materials have been shown to exhibit excellent adsorption and catalytic properties for sulfur species. This unique feature efficiently addresses the notorious shuttle effect and sluggish conversion kinetics of sulfur species, therefore accelerating the bidirectional sulfur conversion process. Among them, oxygen vacancies are the most commonly used anionic vacancies in LSBs, significantly ameliorating the electrochemical performance of LSBs via strengthening charge transfer capacity and improving LiPS affinity [18, 19, 20]. Appropriate oxygen vacancies provide moderate adsorption and facilitate subsequent catalytic conversion, but excess oxygen vacancies will destroy the initial structure, which in turn limits the electrochemical performance [21, 22]. As a result, modulating the interfacial interactions, which are determined by the surface electronic state of the substrate, is extremely critical for heterogeneous redox reactions. Transition metal compounds are one of the most commonly used LiPSs catalysts. In metal‐based compounds containing anions, the electron energy in the p orbital of the non‐metal anion and the d orbital of the metal cation directly influences bond formation and breakage. Moreover, a smaller energy gap between the d‐band and p‐band centers leads to faster interfacial electron transfer kinetics, thereby reducing the reaction energy barrier for Li‐S conversion [23, 24]. This highlights the impact of p‐band and d‐band centers on the electrochemical performance of LSBs [25, 26, 27].

Cobalt tungstate (CoWO_4_), as a transition bimetal oxide, is widely used in electrochemical energy storage applications due to its wolframite structure, excellent electrical conductivity, abundant soil content, multivalent and excellent electrochemical activity [28, 29, 30]. Based on the characteristics of CoWO_4_, it seems that bidirectional catalysis during the charging and discharging of lithium–sulfur batteries can be achieved by adjusting the oxygen vacancy concentration of cobalt tungstate. In this work, CoWO_4‐_ *_x_*/CNT composites with varying concentrations of oxygen vacancies were then prepared by controlling the time of the argon–hydrogen thermal reduction. Consequently, the battery with CWO‐M achieved impressive an excellent rate performance of 768 mAh g^−1^ at 2 C and high capacity retention of 91.1% after 100 cycles at 0.2 C. Stable cycling performance with a areal capacity of nearly 4 mAh cm^−2^ can be retained even after 100 cycles at an extremely high sulfur loading of 8.02 mg cm^−2^ and a low electrolyte/sulfur (E/S) ratio of 8 µL mg S^−1^. Theoretical calculations show that the Li_2_S dissociation energy barrier of CoWO_4−_ *_x_* (0.77 eV) is significantly lower than that of CoWO_4_ (1.16 eV). The introduction of oxygen vacancies improves the conductivity and the adsorption and conversion capacity of the material to LiPSs due to strong p–d orbital hybridization. This work provides valuable insights into the development of highly active catalysts for LSBs by oxygen vacancy regulation.

## 2 Results and Discussion

**FIGURE 1:**
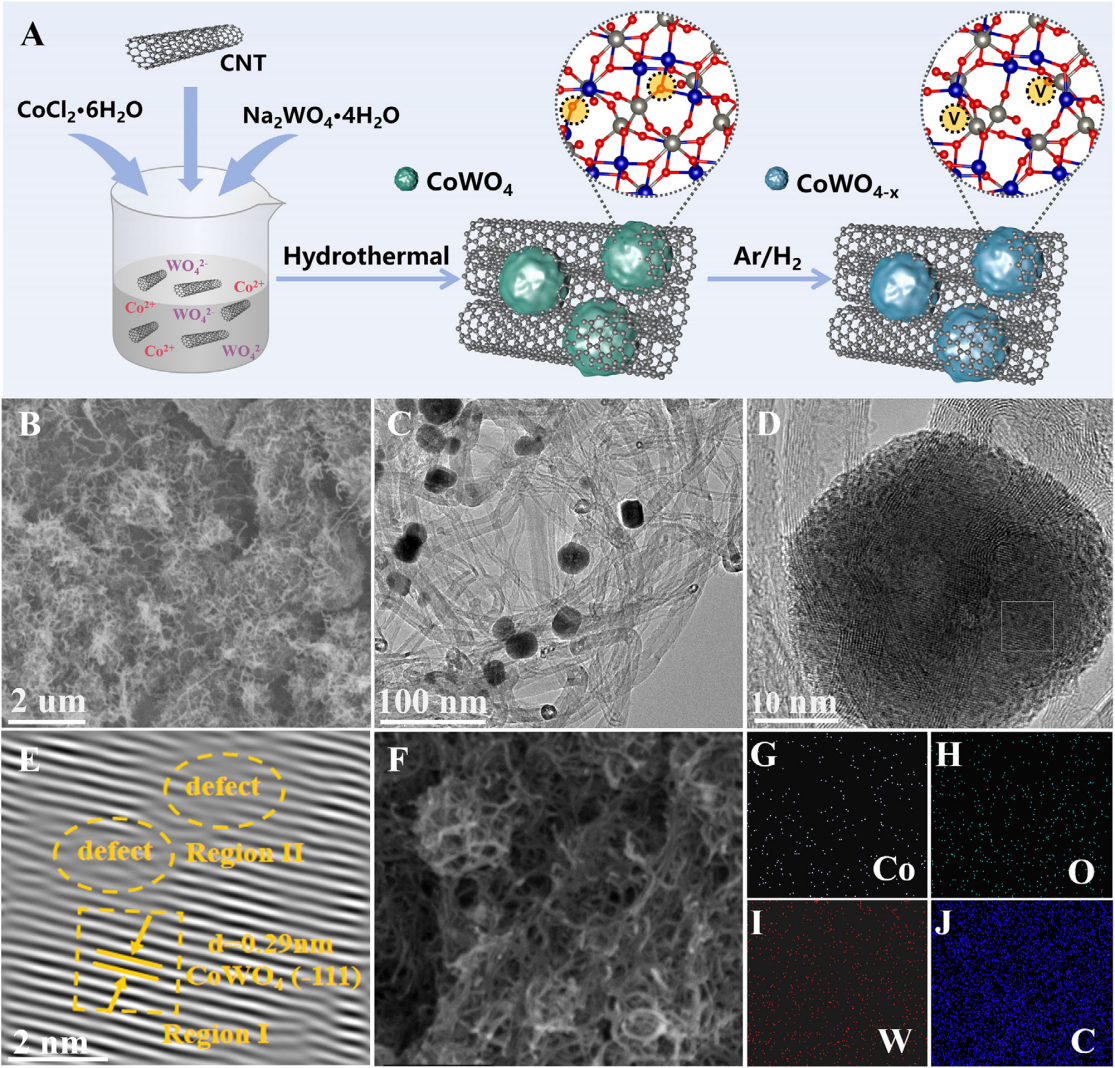
(A) Schematic diagram of the synthesis of CoWO_4−_ *_x_* **/**CNT materials. (B) SEM images. (C) TEM images. (D) HRTEM image of CWO‐M. (E) Inverse IFFT lattice images. (F–J) Elemental mapping images of CWO‐M.

The synthesis process of the sample is displayed in Figure 1A. CoWO_4_/CNT composite materials were first obtained by the hydrothermal method and named CWO. Then, the reduction reaction was conducted in a tube furnace in H_2_/Ar with different times (1, 4, and 6 h) to obtain the product CoWO_4−_ *_x_*/CNT with different oxygen vacancy concentrations, and the final products were named CWO‐L, CWO‐M, and CWO‐H, respectively. As demonstrated in Figure S1, the thermogravimetric analysis test yielded an estimated 30 wt% CoWO_4_ content in the composite materials. The structural morphologies of CWO, CWO‐L, CWO‐M, CWO‐H, and CNTs were characterized by scanning electron microscopy (SEM) (Figure 1B and Figure S2). All made of CNTs entangled to create a conductive porous network structure, and this porous shape improves conductivity by facilitating faster ion diffusion and offering quick pathways for carrier transfer [31]. Besides, the microstructure of the CWO‐M crystals was further characterized using transmission electron microscopy (TEM). As observed from Figure 1C, most CoWO_4_ nanoparticles have uniform morphology with a size of approximately 30 nm, growing on the surface of the CNTs (tube diameter 10–20 nm). Further, the inverse fast Fourier transform (IFFT) lattice diagram (Figure 1E) correlation analysis was carried out for the white‐boxed section of Figure 1D. In detail, the lattice spacing of region Ι is 0.29 nm, which corresponds to the (−111) crystal plane of CoWO_4_ (Figure S3). The lattice fringes in region II are clearly deformed, suggesting the presence of some lattice defects and evidencing the formation of CoWO_4−_ *_x_* vacancies. The formation of defects facilitates the emergence of more active sites and increases the electro‐catalytic activity. In addition, energy‐dispersive spectrometry (EDS) mapping of the CWO‐M presents the homogeneous distribution of Co, O, W, and C elements (Figure 1F–J).

**FIGURE 2:**
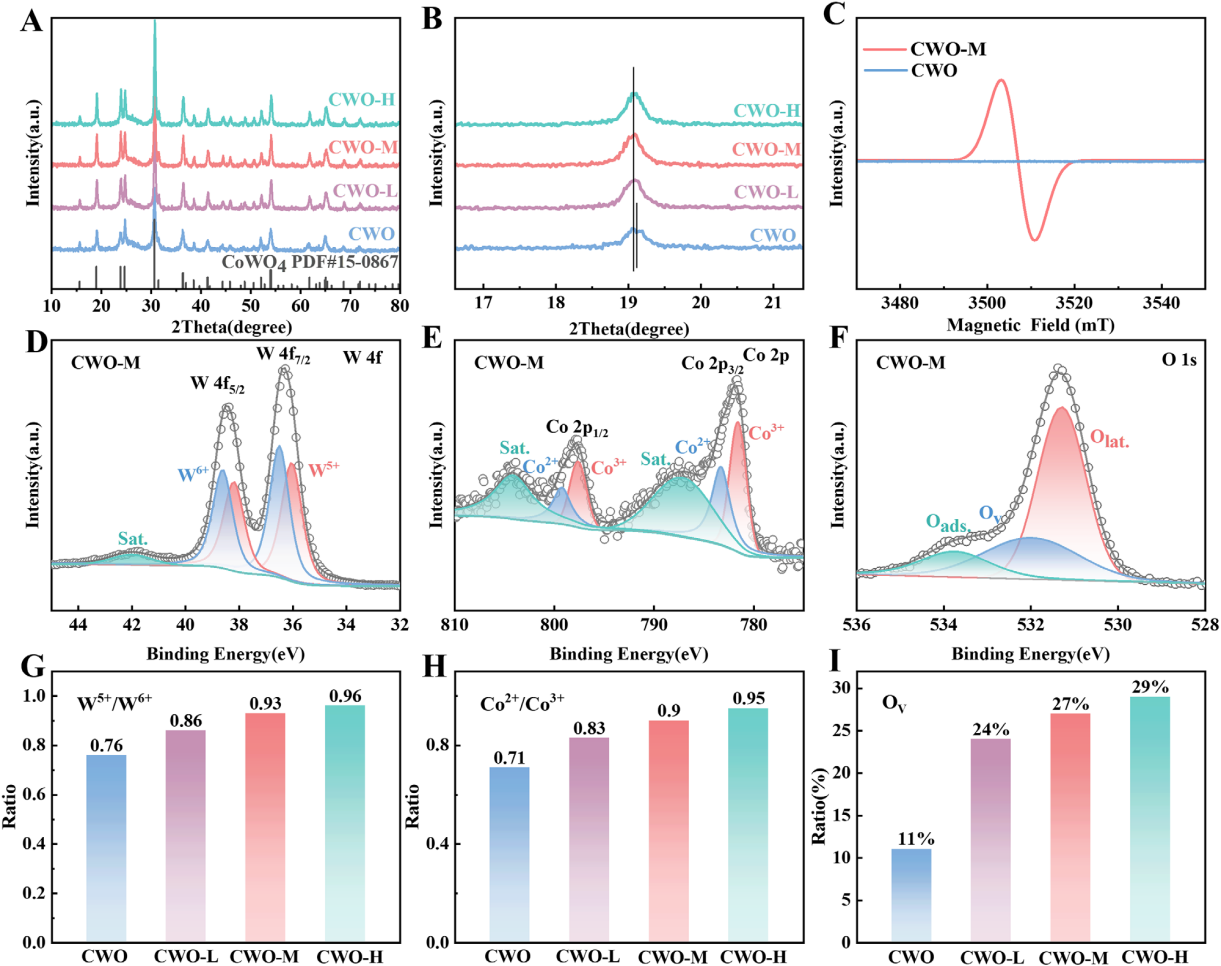
(A,B) XRD pattern of CWO, CWO‐L, CWO‐M, and CWO‐H. (C) EPR spectra of CWO‐M. XPS spectra of CWO‐M: (D) W 4f, (E) Co 2p, and (F) O 1s. (G–I) XPS Quantitative analysis of CWO, CWO‐L, CWO‐M, and CWO‐H.

X‐ray diffraction (XRD) pattern was employed to analyze macro‐structural characteristics of CWO, CWO‐L, CWO‐M, and CWO‐H. All the diffraction peaks of the samples matched well with the wolframite CoWO_4_ (JCPDS No. 15‐0867) (Figure 2A), indicating the successful synthesis of these four samples. Moreover, Figure 2B illustrates the enlarged spectra of the (001) facet. The formation of disordered lattice volumes that may be attributed to oxygen defects is proved by the noticeably negative shifts in the CWO‐L/M/H diffraction peaks [31, 32]. The electron paramagnetic resonance (EPR) technique further confirmed the formation of oxygen vacancies. Unpaired electrons localized in surface oxygen vacancies interact with magnetic dipoles, resulting in a strong resonance signal for CWO‐M at a *g*‐value of 2.003 (Figure 2C). This signal confirms the presence of unpaired electrons associated with oxygen vacancies in CWO‐M [29]. In addition, CWO‐H has a more obvious resonance signal (Figure S4) and larger spins mg^−1^ (Table S1) than CWO‐M. This implies that CWO materials subjected to varying durations of heat treatment exhibit differences in oxygen vacancy concentrations; specifically, prolonged heat treatment results in a higher concentration of oxygen vacancies [33].

X‐ray photoelectron spectroscopy (XPS) is displayed in Figure S5. A doublet peak at 36.3 and 38.5 eV assigned to the W 4f peak was deconvoluted to W^6+^ and W^5+^ species as shown in Figure 2D and Figure S6A, respectively [34, 35, 36]. Compared to CoWO_4_, the W 4f peak of CoWO_4‐_ *_x_* shifts toward lower binding energies, with the shift becoming more pronounced as the vacancy concentration increases. This behavior can be attributed to the presence of oxygen vacancies, which lead to an increase in electron cloud density and a subsequent decrease in the valence state of the tungsten element [20]. Meanwhile, Co^3+^, Co^2+^, and satellite Co species from the deconvoluted Co 2p peak were clearly exhibited in Figure 2E and Figure S6B [28, 29]. As illustrated in Figures 2G,H, the quantitative analysis showed that longer Ar/H_2_ annealing times resulted in more Co^2+^ and W^5+^ species. This increase is attributed to the presence of oxygen vacancies, which leads to a reduction in the oxidation states of the Co and W elements. In addition, the O 1s XPS spectra in Figure 2F and Figure S6C also demonstrate the presence of oxygen vacancy at different concentrations. The oxygen species are composed of lattice oxygen (Olat.), oxygen vacancy (O_V_), and adsorbed oxygen (Oads) [20, 29, 34]. The fitting results of the O 1s spectra in Figure 2I verify that the amount of oxygen vacancies rises with the increase of annealing time, which provides more active sites for SRR and SER.

**FIGURE 3:**
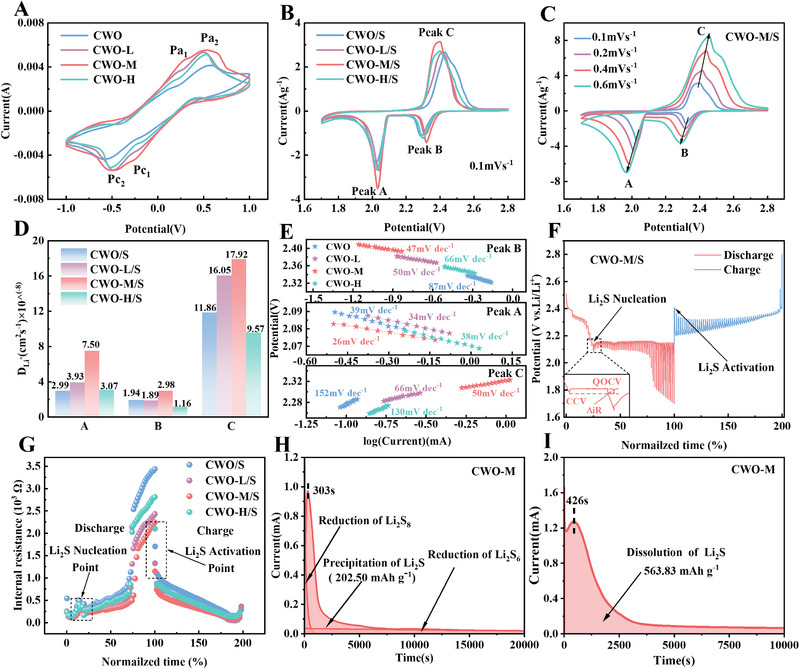
(A) Symmetric cell CV curves for the different cathodes. (B) CV curves at a scan rate of 0.1 mV s^−1^. (C) CVs at various scan ratios of CWO‐M/S. (D) Calculated *D*_Li+_ for electrodes at various stages. (E) Tafel slope evaluation of CV curves of the various cathodes. (F) GITT curves of CWO‐M/S. (G) Internal impedance comparison diagram. (H,I) Potentiostatic discharge/charge curves of Li_2_S_8_ solution on CWO‐M.

Visual adsorption experiments are commonly used to compare the adsorption properties of various catalysts for Li_2_S_6._ As depicted in Figure S7, immersing each material of the same mass in the Li_2_S_6_ solution for 5 h, significant differences between the materials were observed. In detail, CWO‐L becomes colorless, and the color of the supernatant with CWO‐M is almost faded, while the CWO‐H shows minimal color change. This phenomenon can be attributed to the excessive concentration of oxygen vacancies, which disrupts the initial crystal structure and increases local disorder around these vacancies, thereby creating a locally unsaturated environment. These disruptions result in an uneven charge distribution, which significantly affects the p‐orbital electrons of sulfur in Li_2_S_6_. This, in turn, induces stronger local electrostatic repulsion, ultimately weakening the material's adsorption capability [37]. Weak adsorption cannot restrain the shuttling of LiPSs, while too strong adsorption will break the Li─S bond that LiPSs originally formed, which will prevent LiPSs from forming. Therefore, CWO‐M with moderate adsorption ability may have the best catalytic function [38, 39, 40]. Moreover, symmetric cells were assembled with Li_2_S_6_ electrolytes to gauge the kinetics of oxidation and reduction during the liquid–liquid conversion process. Remarkably, CWO‐M exhibited redox peaks and the strongest redox current response in these four samples (Figure 3A), suggesting that CWO‐M has better catalytic performance. Meanwhile, the CWO‐M cell showed two prominent pairs of redox peaks, the two cathode peaks corresponding to the conversion of S_8_ to Li_2_S*_n_* and the conversion of Li_2_S*_n_* to Li_2_S, and the two anode peaks representing the oxidation of Li_2_S to Li_2_S*_n_* and further oxidation to S_8_, significantly enhancing the kinetics of the LiPSs conversion reaction [41, 42]. Additionally, LSBs were assembled with CWO/S and CWO‐L(/M/H)/S cathodes, and the electrocatalytic activity of these cathode materials was further studied. Figure 3B illustrates the CV curves of the batteries with different cathodes. The cathode peak of CWO‐M/S (approximately 2.03 V) showed a higher current density compared to CWO/S, CWO‐L/S, and CWO‐H/S (Figure S8A), suggesting that LiPSs are more easily converted to insoluble Li_2_S_2_/Li_2_S with CWO‐M/S [43]. Significantly, the oxidation peak of CWO‐M/S shifts to a much lower potential than other samples (Figure S8B), demonstrating an effective capacity for oxidation toward Li_2_S_2_/Li_2_S [44].

Lithium‐ion diffusion coefficient (*D*_Li+_) can directly reflect the conversion kinetics of LiPSs [45]. CV measurements of four cathodes at various scan ratios (Figure 3C and Figure S9A–C) are further carried out to calculate the *D*_Li+_ [38]. Compared with other sulfur cathodes, the CWO‐M/S cathode has a stronger peak current and a smaller potential spacing, indicating less electrochemical polarization. Based on the view that the redox peak current varies linearly with the square root of the scan rate, *D*_Li+_ can be estimated using the following Randles–Sevcik Equation (1):

(1) $$I_{p}=2.69\times 10^{5}n^{1.5}AD_{Li^{+}}^{0.5}C_{Li^{+}}V^{0.5},$$

where *I_p_* presents the peak current, *n* is on behalf of the electron number of the redox reaction, *A* is the active area of the electrode, *D*_Li+_ represents the Li^+^ diffusion coefficient, *C*_Li+_ is the Li^+^ concentration, and *v* is the scan rate. The Li^+^ diffusion coefficient has a positive relationship with the slopes of *I*_p_ and *v* ^1/2^ (Figure S9D). The results of the computation indicate that *D*_Li+_ of A, B, and C peaks of the CWO‐M/S electrode are 7.50 × 10^−8^, 2.98 × 10^−8^, and 1.80 × 10^−7^, respectively (Figure 3D), which are larger than those of CWO/S, CWO‐L/S, and CWO‐H/S electrodes. This finding suggests that in LSBs, a modest concentration of oxygen vacancies facilitates ion diffusion and thus bidirectional sulfur conversion processes.

Electrochemical impedance spectroscopy (EIS) measurement was carried out to demonstrate the accelerated conversion kinetics of LiPSs [46] (Figure S10). As the concentration of oxygen vacancies rises, the charge transfer resistance (*R*_ct_) first decreases and then increases. CWO‐M/S electrode exhibits the lowest *R*_ct_ concerning the other electrodes. In general, a defect means a distortion of the perfect crystal structure that disturbs the original electron distribution and forms many active sites by creating dangling bonds, which is beneficial for charge transfer during electrochemical processes [47]. However, due to a significant quantity of structural distortions and dislocations, the transport electron process was hindered by excessive defects [48]. In addition, the conductivity measured in Table S2 also showed a tendency to increase and then decrease with the concentration of oxygen vacancies, corresponding to the electrochemical impedance spectrum, which proved that moderate oxygen vacancies can increase the conductivity, while excessive oxygen vacancies will reduce the conductivity [37].

To further prove the above conclusion, Tafel plots based on CV curves were acquired to assess the impact of different materials on redox kinetics. The CWO‐M/S shows smaller Tafel slopes (Figure 3E) of oxidation and reduction peaks, revealing that moderate oxygen vacancies can accelerate Li_2_S precipitation and LiPSs redox kinetics. The internal resistance of cells with CWO/S, CWO‐L/S, CWO‐M/S, and CWO‐H/S cathodes was further investigated by measuring the galvanostatic intermittent titration (GITT) curve of 0.1C during charging and discharging (Figure 3F and Figure S11). The depth of inclination of the discharge/charge curve represents the internal resistance of Li_2_S nucleation and activation (indicated by the arrows in Figure 3F). The relative size of Δ_Rinternal_ in GITT tests can be used to quantify polarization during the discharge–charge process based on the following relationship:

(2) $$R_{\text{internal}}\left(\Omega \right)=\left|\Delta V_{\text{QOCV-CCV}}\right|/I_{\text{applied}},$$

where Δ*V*_QOCV‐CCV_ is the voltage difference between the points of quasi‐open‐circuit voltage (QOCV) and closed‐circuit voltage (CCV), and *I*_applied_ is the applied current, as depicted in Figure 3F inset [49]. Between the Li_2_S nucleation and activation stages, the CWO‐M/S battery shows lower Δ*R*_internal_ values than the other batteries (Figure 3G), suggesting that the CWO‐M/S cathode has the lowest Δ*R*_internal_. The results of the aforementioned studies demonstrate that the CWO‐M efficiently accelerates the sulfur redox reaction.

To ascertain the reason for the fast catalytic reaction kinetics of CWO‐M with moderate oxygen vacancy concentrations, variable temperature EIS tests were performed on four cathode materials (Figure S12) and the relationship between In(*R* ^−1^) and *T* ^−1^ was plotted (Figure S13A), with *R*_ct_ decreasing with increasing temperature. The apparent activation energy (*E*_a_) of LiPSs was obtained by fitting *R*_ct_ versus temperature according to the Arrhenius equation and calculating the slope, as shown in Figure S13B. The results show that the activation potential of CWO‐M/S (11.11 kJ mol^−1^) electrode is significantly lower than that of CWO/S (17.02 kJ mol^−1^), CWO‐L/S (12.73 kJ mol^−1^), and CWO‐H/S (16.27 kJ mol^−1^) electrodes, indicating faster charge transfer kinetics, which in turn could boost the evolution of sulfur species.

To estimate the significant LiPS reduction and Li_2_S oxidation properties of CWO‐M, the Li_2_S nucleation and decomposition measurement, as another key kinetic indicator for LSBs, was performed [50, 51]. As seen in Figure 3H,I and Figure S14, the initial monotonically decreasing current is connected to the conversion of the remaining higher‐order LiPSs to Li_2_S_4_. The subsequent current peak is attributed to the further nucleation and expansion of solid Li_2_S. The sharp nucleation peak of the CWO‐M cathode is significantly earlier than in other samples, indicating that the solid Li_2_S has a faster nucleation rate (Figure 3H and Figure S14A–C). Furthermore, the nucleation capacity is associated with the integrated area beneath the current curves. Remarkably, the CWO‐M cell provides a significantly specific capacity of 202.50 mAh g^−1^, which is superior to CWO, CWO‐L, and CWO‐H, suggesting the efficient deposition of Li_2_S caused by CWO‐M. The discharge curve of CWO‐M shows the minimum potential dip and also intuitively demonstrates the improvement of Li_2_S precipitation on CWO‐M (Figure S15A). Likewise, Figure 3I and Figure S14D–F show the potentiostatic charge process at 2.4 V, the highest dissolution capacity (260 mAh g^−1^) was found on the CWO‐M catalyst surface, which indicates the effective oxidation of Li_2_S with the CWO‐M catalyst. Moreover, the Li_2_S oxidation process was explained by presenting the charge profiles of several cathodes in Figure S15B. Due to the reduced overpotential, CWO‐M demonstrates enhanced capability in surmounting energy barriers, thus promoting the oxidation of Li_2_S. These all show that during the discharge and charge process, the CWO‐M concurrently encourages Li_2_S nucleation and dissolution.

**FIGURE 4:**
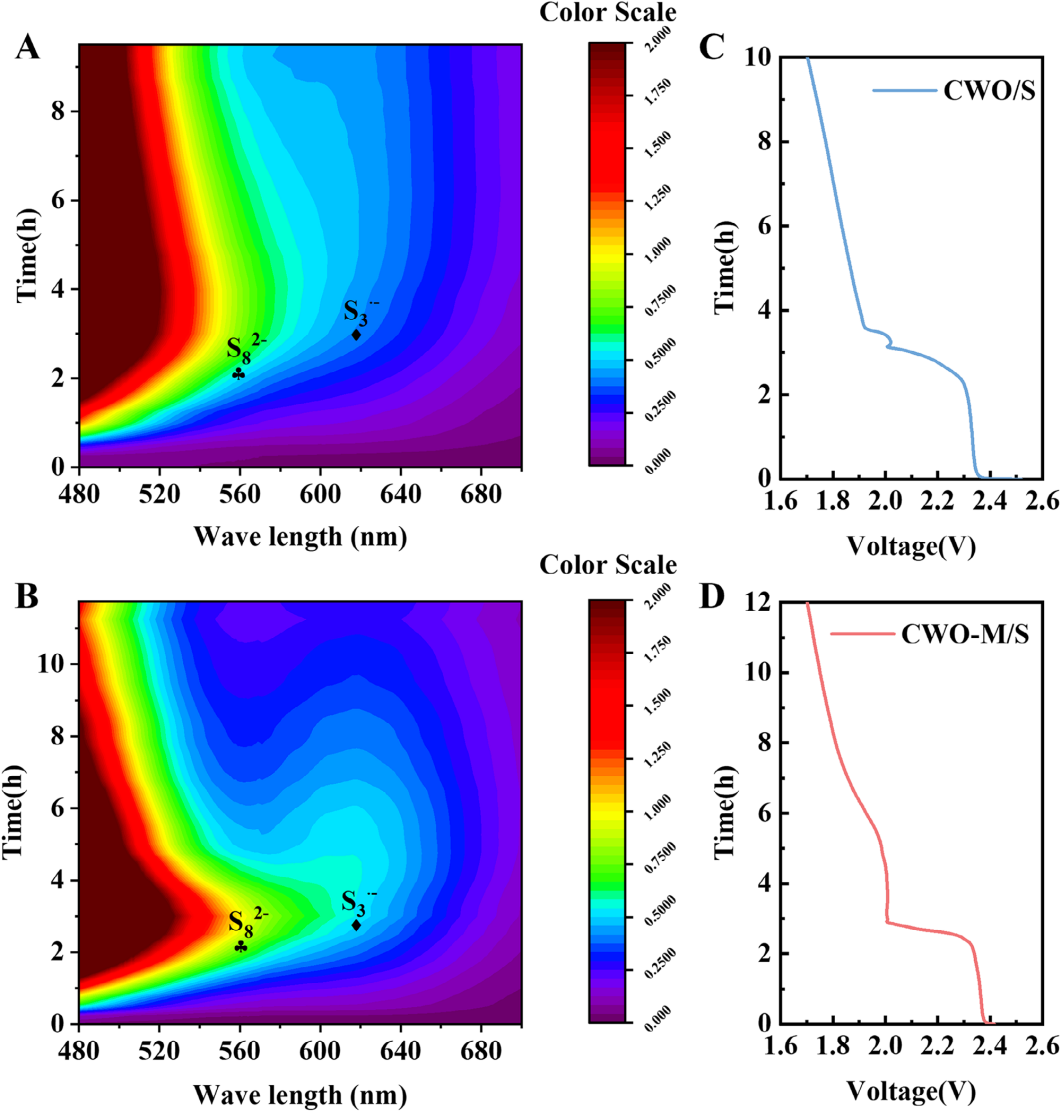
In situ UV–vis spectroscopy of the cell with (A) CWO/S and (B) CWO‐M/S cathodes; (C,D) Corresponding galvanostatic discharge curves of (A,B).

To better understand the mechanism of rapid LiPSs conversion in LSBs with CWO‐M as the catalyst, in situ UV–vis spectroscopy was employed during the discharging process of LSBs (Figure 4A,B). The corresponding discharge curves demonstrate that the CWO‐M/S electrode has a longer discharge time than the CWO/S electrode (Figure 4C,D). The spectra of the CWO‐M/S electrodes reveal higher concentrations of S8^2−^, which can most likely be attributed to the redox reactions occurring between CWO‐M/S and LiPSs. Concurrently, the concentration of trisulfur radical (S_3_^•−^) was consistently greater on the CWO‐M/S electrode. This is explained by the fact that CWO‐M catalysts effectively promote the dissociation process of S_6_^2−^ (S_6_^2−^ → 2S_3_^•−^). Further consumption of solid sulfur by the S_3_^•−^ radical could result in the formation of further electrochemically reducible LiPSs (2S_3_^•−^ + 1/4S_8_ → S_8_^2−^) [52]. The S_3_^•−^ is able to keep a steady, elevated concentration in the in situ visual vial cell system with a CWO‐M/S electrode. This suggests that the S_3_^•−^ produced by the dissociation of S_6_^2‐^ can efficiently encourage the opening of the S_8_ ring, indicating favorable sulfur utilization and rapid electrochemical reaction kinetics of LSBs. To summarize, in situ UV–vis spectroscopy has shown that CWO‐M catalysts can facilitate the SRR process by means of the accelerated dissociation of Li_2_S_6_ into trisulfur radical species (S_3_^•−^) in LSBs, which further evidence that the presence of oxygen vacancies is able to efficiently promote the conversion of LiPSs.

**FIGURE 5:**
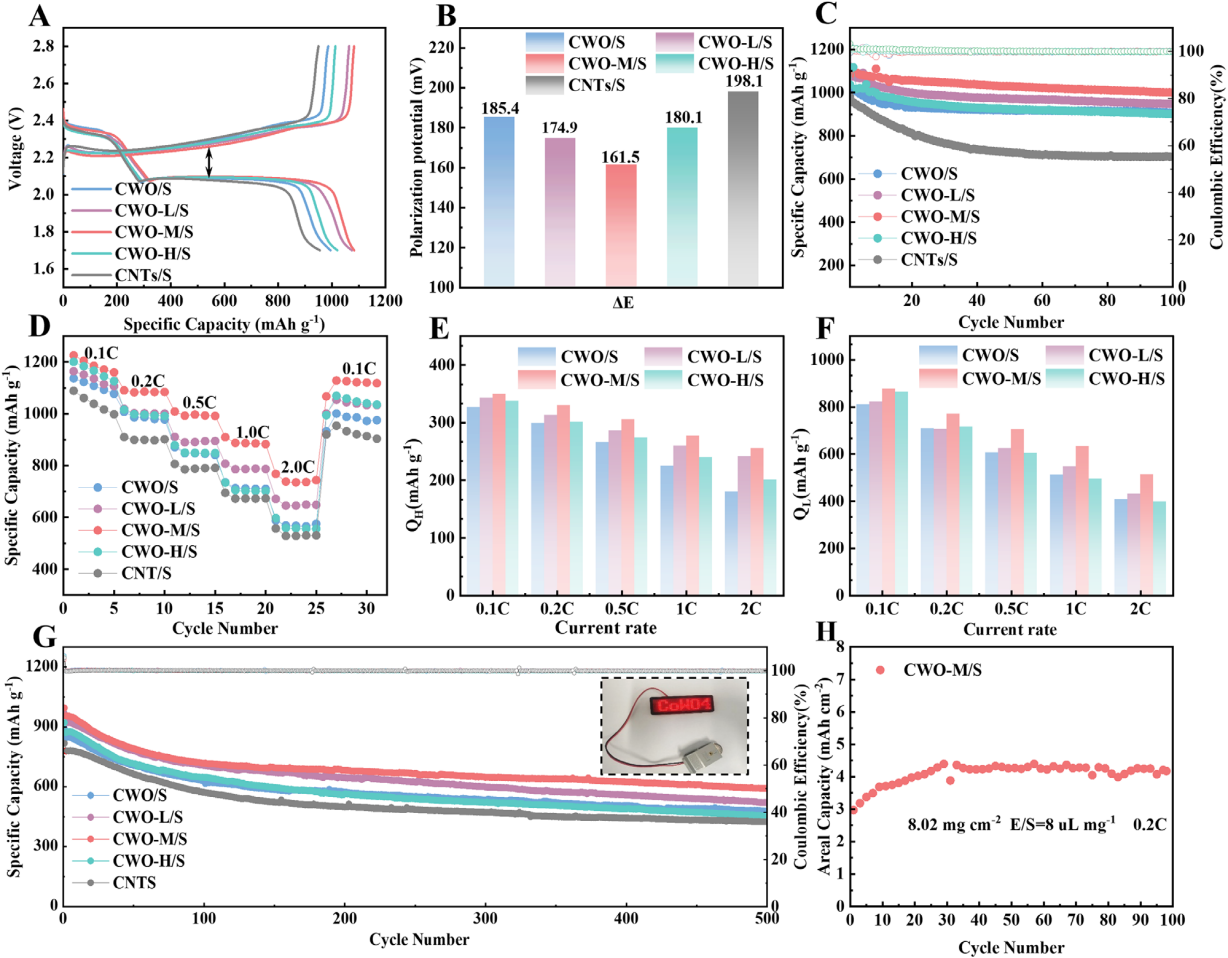
(A) The discharge–charge curves of various cathodes at 0.2 C and (B) Δ*E* values derived from charge‐discharge curves. (C) The cycling performance at 0.2 C of various cathodes. (D) The rate performances of various cathodes. (E) *Q*_H_ and (F) *Q*_L_ of charge–discharge curves at different rates of various cathodes. (G) Long‐term cycle performance at 1 C and digital images of LED illuminated by CWO‐M/S. (H) The cycling performance of a LSB with a sulfur loading of 8.02 mg cm^−2^.

Various electrochemical performance tests were conducted to investigate the function of CWO/CWO‐L/M/H for LSBs. First, to understand the self‐discharge behavior of LSB, a test of the resting voltage was carried out (Figure S16). Cells consisting of different materials were rested on the test channel, and then the value of the open circuit voltage as a function of time was recorded. The CWO‐M/S cell has the largest stable value of open circuit voltage after 5000 min, indicating that CWO‐M efficiently suppresses the shuttling of long‐chain sulfur species, thus alleviating the self‐discharge behavior of LSBs. The CWO‐M/S electrode has a comparatively lower polarization, as seen by the galvanostatic charge–discharge curve (GCD) data (Figure 5A), and its Δ*E* value is solely 161.5 mV, while the values for the CWO/S (185.4 mV), CWO‐L/S (174.9 mV), CWO‐H/S (180.1 mV), and CNTs/S (198.1 mV) electrodes are all larger, as depicted in Figure 5B. This implies that catalytic material with the right amount of oxygen vacancies lowers the energy barrier for reactions and accelerates the SRR and SER process of LSBs. Besides, the CWO‐M/S electrode shows a remarkable capacity of 1096.1 mAh g^−1^ at 0.2 C and keeps its capacity of 998.7 mAh g^−1^ after 100 cycles retaining the capacity of 91.1%, superior to the CWO/S (904.8 mAh g^−1^, 87.3%), CWO‐L/S (946.1 mAh g^−1^, 84.5%), CWO‐H/S (900.4 mAh g^−1^, 86.9%) and CNTs/S (703.2 mAh g^−1^, 71.7%) (Figure 5C). The rate performance of different batteries is depicted in Figure 5D. The CWO‐M/S cathode exhibits exceptional rate performance with capacities of 1225(0.1 C), 1090(0.2 C), 1008(0.5 C), 909(1 C) and 768 mAh g^−1^(2 C). When returning to 0.1 C, the capacity of the CWO‐M/S cathode can be restored to 1067 mAh g^−1^, revealing that the existence of CWO‐M/S catalyst can maintain the stability of the cathode structure under high current rates.

Subsequently, the charging/discharging profiles of the CWO/S, CWO‐L/S, CWO‐M/S, and CWO‐H/S electrodes are analyzed in detail (Figure S17). Even at a high current rate of 1 C, the CWO‐M/S cathode still behaves an adequate performance. As shown in Figure 5E,F, CWO‐M/S exhibits the longest high potential discharge plateau (*Q*_H_) and low potential discharge plateau (*Q*_L_), and the maximum discharge capacitance. This further suggests that CWO‐M/S is an efficient catalyst for the conversion of S_8_ to LiPSs and then to Li_2_S. In the high potential range (2.1–2.4 V), the discharge capacity increases when oxygen vacancies are present and increases first and then decreases as the oxygen vacancy concentration increases. Furthermore, the capacity retention rate of different rates relative to 0.1 C (Figure S18A) is similarly in line with this trend, which further illustrates that appropriate oxygen vacancies have better catalysis and stability. Within the range of low potential (1.7–2.1 V), although the occurrence of oxygen vacancies improved the catalytic activity, the high oxygen vacancy concentration weakened the adsorption for LiPSs and hindered their reduction process, so that CWO‐H/S at high magnification had the lowest *Q*_L_. Meanwhile, the high concentration of oxygen vacancies decreases the conductivity, and as the magnification increases, the polarization increases, resulting in the lowest capacity retention of CWO‐H (Figure S18B).

Following an activated process with a low current rate (0.1 C), the discharge capacity at a high current rate (1 C) can reach 994.6 mAh g^−1^, and a reversible capacity of 592.2 mAh g^−1^ can be maintained after 500 cycles, with a capacity decay of only 0.081% each cycle (Figure 5G). In contrast, CWO/S, CWO‐L/S, CWO‐H/S, and CNTs/S cathodes deliver initial discharge capacities of 893.8, 966.4, 921.1, and 818.2 mAh g^−1^, respectively. During the cycle performance test, their capacity decay rates are also larger than those of CWO‐M/S. In addition, Table S3 shows the comparison of the electrochemical performance of cathode materials containing vacancy structures in LSBs with CWO‐M in recent years, further demonstrating its superior electrochemical performance. As shown in Figure 5H and Figure S19, the performance of the CWO‐M/S cathode is also satisfactory when the sulfur loading is increased to 6.17 and 8.02 mg cm^−2^ and low (E/S) ratios, with a surface capacity of more than 4 mAh cm^−2^ at either 0.1 C or 0.2 C with a low E/S ratio, meeting industrial needs. To demonstrate the usefulness of LSBs, Figure 5G illustrates a 2025 type cell equipped with CWO‐M/S may illuminate light‐emitting diodes (LEDs) that display the cobalt tungstate. The CWO‐M/S exhibits exceptional rate performance, cycle stability, and high areal capacity, suggesting that its appropriate concentration of oxygen vacancy could serve as an efficient electrocatalyst for the advancement of LSBs.

**FIGURE 6:**
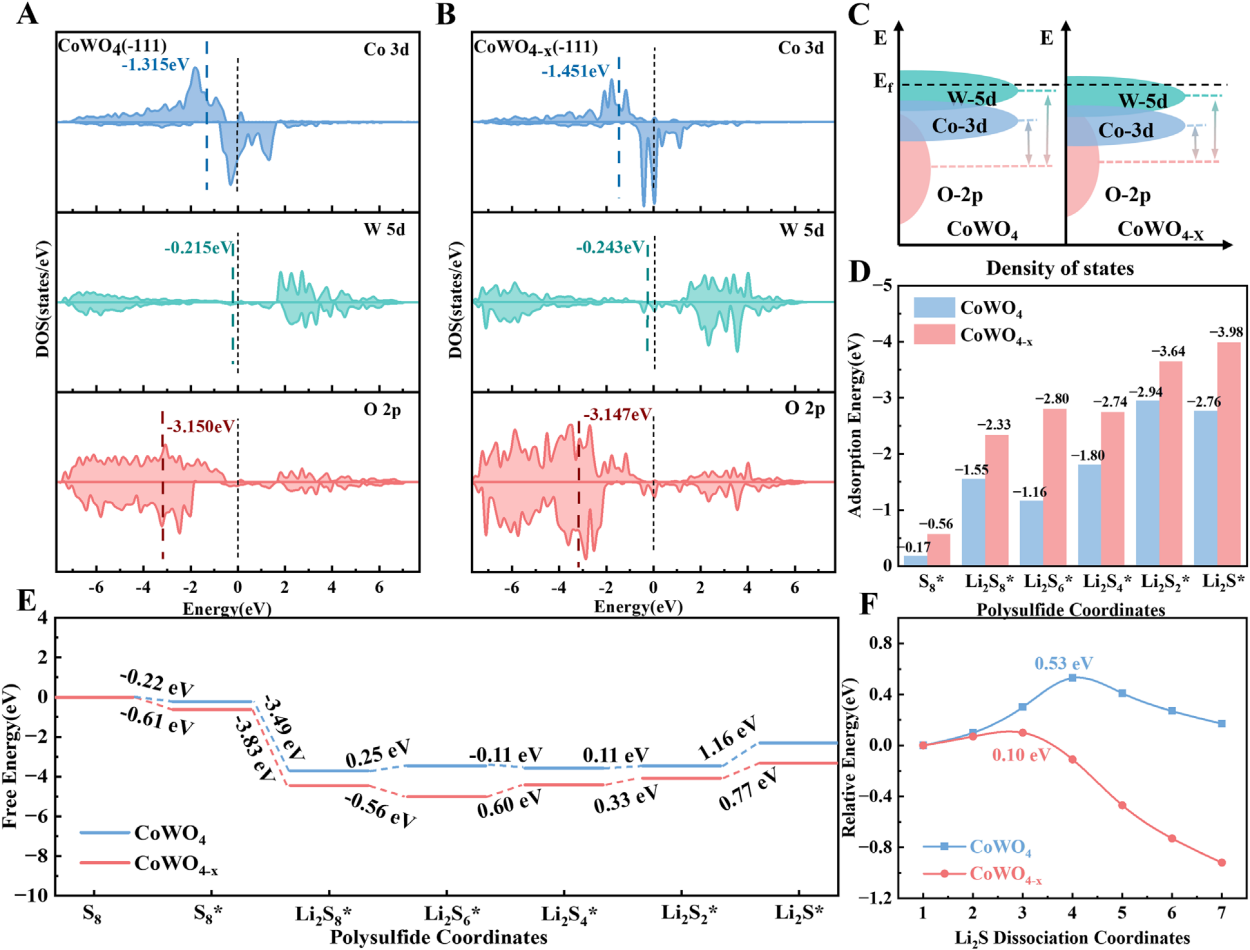
The projected partial density of states of (A) CoWO_4_ and (B) CoWO_4−_ *_x_*. (C) Diagrammatic representation of d‐band and p‐band changes following vacancy correction. (D) Theoretical calculations of the binding energies between the catalysts and LiPSs. (E) Free energies of CoWO_4_ and CoWO_4−_ *_x_* during discharge from S_8_ to Li_2_S. (F) Decomposition barriers of Li_2_S on CoWO_4_ and CoWO_4−_ *_x_*.

Through theoretical computation, the impact of oxygen vacancies on the electrical structure of CoWO_4_ was examined (Figure 6). The mechanism of oxygen vacancies on the adsorption and catalytic performance of LSBs was also clarified. Figure 6A,B and Figure S20 display the projected total/partial density of states of CoWO_4_ and CoWO_4−_ *_x_*. Compared to CoWO_4_, more electronic states stay near/across the Fermi level of CoWO_4−_ *_x_*. This illustrates that more oxygen vacancies can improve the conductivity and achieve better electrochemical performance [18, 29]. Moreover, Co, W, and O elements showed a great hybridization with the existence of O vacancies (Figure 6C). The centers of the Co 3d band and the W 5d band of CoWO_4−_ *_x_* move downward in comparison to CoWO_4_, while the center of the O 2p band moves upward. This results in a decrease in the energy gap between the center of the d band and the center of the p‐band, thereby enhancing charge transfer and reducing the activation energy barrier [26, 27]. Figure 6D calculates the binding energies between sulfur species and catalysts, and Figures S21 and S22 display the corresponding optimal adsorption configurations. The CoWO_4−_ *_x_* exhibits a stronger adsorption ability for sulfur species than CoWO_4_. Therefore, the CoWO_4−_ *_x_* can effectively reduce the shuttling of LiPSs. Figure 6E illustrates the free energies of the products resulting from the stepwise disproportionation of CoWO_4_ and CoWO_4−_ *_x_* during the sulfur transformation reaction. The results indicate that the final step is the one that determines the rate of the evolution of sulfur species because the reduction from Li_2_S_2_ to Li_2_S has the largest positive Gibbs free energy throughout the whole reduction process. Crucially, the reduction of sulfur species is thermodynamically more advantageous on CoWO_4−_ *_x_* than CoWO_4_, as evidenced by the fact that the value of CoWO_4−_ *_x_* (0.77 eV) is clearly lower than that of CoWO_4_ (1.16 eV). Meanwhile, the decomposition energy of Li_2_S on CoWO_4_ and CoWO_4−_ *_x_* was calculated (Figure 6F). During reverse charging, the Li_2_S decomposition potential (0.10 eV) on the surface of CoWO_4−_ *_x_* was lower than CoWO_4_ (0.53 eV), revealing that the Li─S bond is easily dissociated in the existence of oxygen vacancies, resulting in rapid delithiumation kinetics, further revealing the positive effect of CoWO_4−_ *_x_* on Li_2_S decomposition during oxidation. The aforementioned computations show that the introduction of oxygen vacancies modulates the electronic structure of CoWO_4_, increases the adsorption sites and improves the catalytic activity. Moreover, the bidirectional role of CoWO_4−_ *_x_* in LSBs is also verified, which is consistent with the experimental data.

## 3 Conclusion

To summarize, the CoWO_4−_ *_x_*/CNT composites for sulfur cathodes were successfully synthesized, and it was revealed that CWO‐M with an appropriate amount of oxygen vacancies could accelerate the bidirectional conversion of sulfur species. The Oxygen vacancies not only provide additional active sites for the adsorption and catalysis of LiPSs, but also promote the precipitation and dissolution of Li_2_S. Kinetic experiments and DFT calculations have demonstrated that CWO‐M has a most efficient bidirectional catalytic effect on the conversion between solid sulfur, soluble LiPSs and solid Li_2_S due to the strong p–d orbital hybridization. As a result, reliable and fast catalytic conversion is achieved, yielding remarkable cyclability, with an excellent rate performance of 768 mAh g^−1^ at 2 C and a capacity retention rate of 91.1% after 100 cycles at 0.2 C. In addition, stable cycling performance with a high capacity of nearly 4 mAh cm^−2^ was achieved even after 100 charge/discharge cycles at a high sulfur loading of 8.02 mg cm^−2^ and a low E/S ratio of 8 µL mg S^−1^. Overall, this work enables a simple bidirectional catalyst design through vacancy engineering and contributes to the understanding of the catalytic mechanism of catalysts with different oxygen vacancy concentrations in LSBs.

## Author Contributions

**Yan Chen**: writing – original draft, investigation, methodology, data curation. **Dan Li**: writing – original draft, investigation, methodology. **Yufang Chen**: visualization, conceptualization. **Xingqiao Wu**: conceptualization, writing – review and editing. **Manfang Chen**: resources, funding acquisition. **Yuchao Du**: software, formal analysis. **Keyang Fu**: data curation, formal analysis. **Hao Yuan**: methodology, writing – review and editing. **Shuangying Wei**: methodology, conceptualization. **Xianyou Wang**: supervision, resources. **Hongbo Shu**: conceptualization, project administration, funding acquisition, supervision, writing – review and editing.

## Conflicts of Interest

The authors declare no conflicts of interest.

## Supporting information




**Supplementary File 1**: exp270084‐sup‐0001‐SuppMat.docx.

## Data Availability

The data that support the findings of this study are available from the corresponding author upon reasonable request.
