# Supplementary material for: Enhancing Bidirectional Sulfur Conversion Through p–d Orbital Hybridization via Vacancy Engineering
Source: Exploration (Beijing). 2025 Aug 26;5(6):20240362. doi: 10.1002/EXP.20240362 (PMC12752616; doi:10.1002/EXP.20240362)
Supplement: Supplementary file 1 — exp270084‐sup‐0001‐SuppMat.docx. [file EXP2-5-20240362-s001.docx]

Supporting Information

**Enhancing Bidirectional Sulfur Conversion through p-d Orbital Hybridization via Vacancy Engineering**

*Yan Chen^#a^, Dan Li^#a^, Yufang Chen^#b^, Xingqiao Wu^*c^, Manfang Chen^*a^, Yuchao Du^a^, Keyang Fu^a^, Hao Yuan^d^, Shuangying Wei^e^, Xianyou Wang^a^, Hongbo Shu^^[[1]](#footnote-1)^*a^*

*^a^ Hunan Province Key Laboratory for Electrochemical Energy Storage and Conversion, National Base for International Science & Technology Cooperation, National Local Joint Engineering Laboratory for Key Materials of New Energy Storage Battery, Key Laboratory of Environmentally Friendly Chemistry and Applications of Ministry of Education, School of Chemistry, Xiangtan University, Xiangtan, 411105, China*

*^b^ College of Aerospace Science and Engineering, National University of Defense Technology, Changsha 410000, China*

*^c^ Institute for Carbon Neutralization Technology, College of Chemistry and Materials Engineering, Wenzhou University, Wenzhou, Zhejiang 325035, China*

*^d^ ZTE Corporation, Shenzhen 518063, P.R.China*

*^e^ Department of Industrial Chemistry, University of Bologna, Campus Navile, Via Piero Gobetti 85, Bologna 40139, Italy*

**Experimental Section**

**1.1 Materials Preparation**

**1.1.1 Preparation of CoWO_4_/CNT (CWO)**

Composites of CoWO_4_ with CNT were synthesized hydrothermally. CoCl_2_·6H_2_O (0.238 g, 1.0 mmol) was dissolved in distilled water (10 mL), followed by nitric acid-treated multi-walled carbon nanotubes (500 mg) and sonicated for 1h. Under stirring at room temperature, distilled water (10 mL) containing Na_2_WO_4_·4H_2_O (0.33 g, 1.0 mmol) was added dropwise to the above solution. The resulting suspension was transferred to a stainless steel autoclave lined with PTFE, kept at 160°C for 12h, and then allowed to cool to room temperature. The precipitate was centrifuged, washed alternately with an excess of distilled water and anhydrous ethanol and finally dried at 60°C to give CoWO_4_/CNT, named CWO.

**1.1.2 Preparation of CoWO_4-x_/CNT**

The CoWO_4_/CNT complexes were calcined at 600°C at a heating rate of 5°C min^-1^ under a 5% Ar/H_2_ atmosphere for 1h, 4h and 6h to obtain products containing different oxygen vacancy concentrations as CWO-L, CWO-M and CWO-H, respectively.

**1.1.3 Preparation of** **CWO/S,** **CWO-L/S, CWO-M/S and CWO-H/S composites**

Weigh CWO and S with a mass ratio of 4:6, grind them for 30 min and put them into a PTFE liner (25mL). CWO/S composites were then prepared by constant heating at 155℃ in an Ar atmosphere for 12h. CWO-L/S, CWO-M/S and CWO-H/S composites were obtained in the same way.

**1.2 Characterization of the materials**

The crystal structure of the as-synthesized materials were examined by X-ray diffraction (XRD, Rigaku, Ultima IV with D/teX Ultra with CuKα radiation). The morphological and detailed structure characterization was investigated by scanning electron microscopy (SEM Carl Zeiss, SIGMA, HD-01-61, Germany) and transmission electron microscopy (TEM, FEI-Tecnai G2 TF20, America). The surface elemental states of as-synthesized materials were characterized by X-ray photoelectron spectra (XPS, Kratos Axis Ultra DLD, Japan). TGA (Series Q50 instrument, USA) was performed to determine the weight ratio of the components in the samples.

**1.3 Electrochemical Measurements**

First of all, the active material (CWO/S, CWO-L/S, CWO-M/S and CWO-H/S), Super P, and polyvinylidene fluoride (PVDF) were evenly mixed at a mass ratio of 7:2:1, and NMP was added as a solvent and stirred for 4 h. After that, the slurry was evenly coated on the carbon foil using a scraper, placed in an air blast oven for 1 h, then transferred to a 50°C vacuum oven for overnight drying, and then cut into small discs. The sulfur mass load was maintained at about 1.0-1.2 mg cm^-2^. The cells were assembled in a glovebox filled with argon. The electrolyte was 1 M LiTFSI solution dissolved in a mixture of dimethoxy ethane and 1,3-dioxolane (1:1, v/v) and 2 wt.% LiNO_3_. The cyclic voltammetry (CV) data were acquired using the electrochemical workstation (DH 7006, Donghua Analytical) in the range 1.7-2.8 V (vs Li/Li). The electrochemical impedance spectroscopy (EIS) measurement was conducted at the open circuit potential and a frequency range of 10^-2^ to 10^5^. Galvanostatic intermittent titration (GITT) test: constant current pulse at 0.1C for 10 minutes and 60 minutes on hold. The galvanostatic charge-discharge (GCD) curves were performed in the charge-discharge voltage range of 1.7-2.8 V at the current density of 0.1-2 C on a Neware tester (BTS 4008T Neware, Shenzhen, China).

**1.4 Theoretical Computation**

Based on density functional theory, the Vienna Ab initio Simulation Package (VASP)^[1-4]^ is used to complete all structural optimization and energy calculation processes. Electron exchange correlations and ion-electron interactions were described by the gradient-corrected Perdew-Burke-Erzerhof (PBE) functions^[5]^ and the projector augmented wave (PAW) method^[6]^, respectively. The cutoff energy was set to 500 eV, and the total energy and force convergence for structure optimization were set to 10^-5^ and 0.01 eV/Å, respectively. A 2×2×1 k-point mesh is used in the calculation process. To consider van der Waals force interactions, a semi-empirical correction for DFT-D3^[7]^ was used. A 2 × 2 supercell in the x-y plane was used, and a 15 Å vacuum layer was set in the z direction to prevent interactions due to periodic repetitive structures. The decomposition energy barrier of Li_2_S was calculated using the Climbing Image Nudged Elastic Band (CI-NEB)^[8]^ method. The corrections for zero-point energy and entropy were calculated using the VASPKIT^[9]^ code in the calculation of the Gibbs free energy. Using the computational hydrogen electrode (CHE)^[10]^ model, the Gibbs free energy change (ΔG) for each elementary reduction step was defined as ∆G = ∆E + ∆ZPE - T∆S, where ΔE represents the reaction energy obtained from the DFT calculation. ΔZPE and ΔS are the changes of zero-point energy and the entropy correction, respectively. T represents the room temperature of 298.15 K. The adsorption energy (E_ads_) of an adsorbed molecule is defined as E_ads_ = E(substrate-Li_n_S_m_) - E(substrate) - E(Li_n_S_m_), where E(substrate-Li_n_S_m_), E(substrate), and E(Li_n_S_m_) represent the energy of Li_n_S_m_ adsorbed on the substrate, substrate, and Li_n_S_m_, respectively.

**1.5 Adsorption tests of Lithium Polysulfide**

The Li_2_S_6_ solution was synthesized by mixing sulfur and lithium sulfide (Li_2_S) with a molar ratio of 5:1 in 1,2-dimethoxymethane/1,3-dioxolane solution (DME/DOL, v/v = 1/1) under continuous stirring in an argon glovebox. The same mass of CWO, CWO-L, CWO-M and CWO-H was infiltrated in 2 mL Li_2_S_6_ solution, respectively, and the color changes can be observed after 20 h.

**1.6 In Situ UV–Vis Measurement**

The samples (CWO/S and CWO-M/S), Super P, and PVDF were mixed uniformly at a mass ratio of 7:2:1 as the cathode material, and nickel foam was selected as the collector (1×1 cm^2^), and the sulfur area mass loading on the cathode electrode was approximately 5.0 mg cm^-2^. Assemble the in situ UV-vis cells in an Ar-filled glove box, first, install the anode (Li metal) and cathode (CWO/S and CWO-M/S) in a custom-made in situ cuvette, and subsequently, add 3 mL of Li-S electrolyte (1.0 mol L^-1^ LiTFSI, in DOL/DME, with 2wt% LiNO_3_) and seal. The assembled in situ cuvettes were placed in a UV-Vis spectrophotometer and discharged at 0.05 C (1C = 1675 mAh g^-1^), and UV-vis data were acquired every 15 minutes.

**1.7 Assembly of the symmetric cell**

The symmetric cell cathodes were prepared by simply mixing the active material and PVDF (mass ratio = 8:2) in NMP. The slurry material was then coated on a piece of Al foil and dried at 60 ℃ for 12 h. The dried material-coated Al foil was cut into some pieces with a diameter of 1 cm. For the symmetric electrochemical measurements, CR2025 coin-type cells with two identical electrodes as the working and counter electrodes and Celgard 2400 as the separator were assembled in a glovebox. The electrolyte in the symmetric cells is 20 µL Li_2_S_6_ solution. The cells were then executed on an electrochemical workstation (CHI 660E, Chenhua Instruments Co., Ltd. China) with a scan rate of 10 mV s^-1^ from -1.0 to 1.0 V.

**1.8 Li_2_S nucleation and decomposition tests**

The Li_2_S_8_ solution was synthesized by mixing sulfur and lithium sulfide (Li_2_S) with a molar ratio of 7:1 in 1,2-dimethoxymethane/1,3-dioxolane solution (DME/DOL, v/v = 1/1) under continuous stirring at 60 C for 12 h to form a Li_2_S_8_ solution. The CWO, CWO-L, CWO-M and CWO-H electrodes for Li_2_S nucleation/decomposition study were prepared similarly to the symmetric cell experiments. And lithium foil was used as the counter electrode with Celgard 2400 separating the working and counter electrodes. 16 µL Li_2_S_8_ solution (0.5 mol L^-1^) as catholyte was added on the cathode side, and 16 µL DME/DOL (v/v = 1/1) solution as the anolyte was dropped on the anode side. For the Li_2_S nucleation and growth measurement, the assembled cells were first galvanostatically discharged at 0.05 C until the potential reached 2.06 V to consume most of the high-order polysulfides and then were potentiostatically discharged at 2.05 V to induce the nucleation and growth of Li_2_S until the current decreased to 10^-5^ A. For the Li_2_S dissolution measurement, the cells were first galvanostatically discharged at 0.05 C until the voltage decreased to 1.7 V to generate Li_2_S and then were potentiostatically charged at 2.4 V for the dissolution of Li_2_S into LiPSs until the current was below 10^-5^ A.

**Supplementary figures**

**Figure S1.** TG curve of various materials.

**Figure S2.** SEM images of (A) CNTs, (B) CWO, (C) CWO-L, (D) CWO-H.


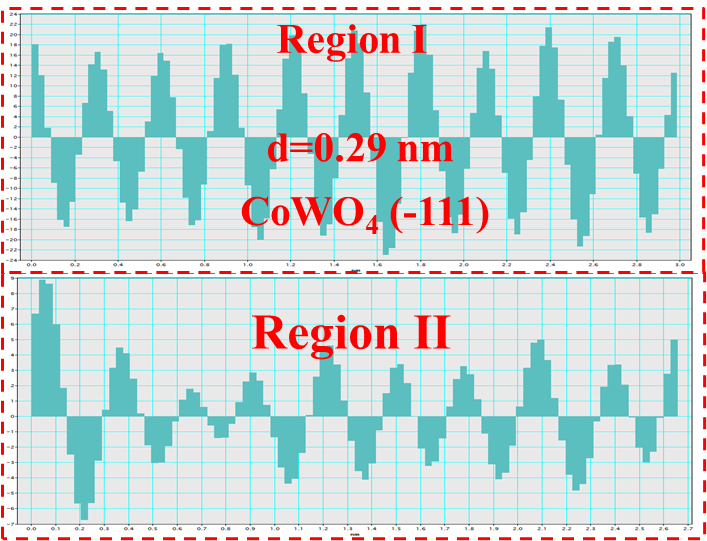


**Figure S3.** Inverse FFT lattice images and corresponding line profiles.

**Figure S4.** EPR spectra of CWO-H.

**Figure S5.** The XPS survey of various materials.

**Figure S6.** XPS spectra of CWO and CWO-L/M/H: (A) W 4f, (B) Co 2p, and (C) O 1s.

**Figure S7.** Adsorption experiments stand for 0h and 5h.

**Figure S8.** Magnified (A) cathodic peak at 2.05 V and (B) anodic peak.

**Figure S9.** CVs at various scan ratios of (A) CWO/S, (B) CWO-L/S, (C) CWO-H/S, (D) linear fitting of peak currents versus square root scan rate of the various cathodes.

**Figure S10.** EIS profiles of four cathodes.

**Figure S11.** Galvanostatic intermittent titration technique (GITT) curves of (A) CWO/S, (B) CWO-L/S, (C) CWO-H/S.

**Figure S12.** EIS curves of (A) CWO/S, (B) CWO-L/S, (C) CWO-M/S and (D) CWO-H/S at different temperatures.

**Figure S13.** (A) Linear fitting of ln(R^-1^) versus 1000 T^-1^ and (B) the comparison of activation energies (Ea) of the various cathodes.

**Figure S14.** Potentiostatic discharge curves of Li_2_S_8_ solution on (A) CWO, (B) CWO-L, (C) CWO-H at 2.05 V; Potentiostatic charge curves on (D) CWO, (E) CWO-L, (F) CWO-H at 2.4 V.

**Figure S15.** Magnifed (A) discharge and (B) charge curves of various cathodes at 0.2 C.

**Figure S16.** Self-discharge behavior of the cells with different cathodes.

**Figure S17.** Charge-discharge curves at different rates of (A) CWO/S, (B) CWO-L/S, (C) CWO-M/S, (D) CWO-H/S.

**Figure S18.** Various rates for different samples relative to 0.1 C capacity retention, (A)Q_H,_ (B)Q_L_.

**Figure S19.** Cycling performance of CWO-M/S with high sulfur loading.

**Figure S20.**The projected total density of states of (A) CoWO_4_ and (B) CoWO_4-x_.

**Figure S21.** The adsorption models and the corresponding adsorption energy value of S_8_, Li_2_S_8_, Li_2_S_6_, Li_2_S_4_, Li_2_S_2_ and Li_2_S on the surface of CoWO_4_.

**Figure S22.** The adsorption models and the corresponding adsorption energy value of S_8_, Li_2_S_8_, Li_2_S_6_, Li_2_S_4_, Li_2_S_2_ and Li_2_S on the surface of CoWO_4-x_.

**Table S1.** Spin concentrations per unit mass of CWO-M and CWO-H.

| **samples** | **CWO-M** | **CWO-H** |
| --- | --- | --- |
| **Spins mg^-1^** | **1.01×10^12^** | **1.86×10^12^** |

**Table S2.** Conductivity of different samples.

| **samples** | **CWO** | **CWO-L** | **CWO-M** | **CWO-H** |
| --- | --- | --- | --- | --- |
| **10^-5^ σ (S cm^-1^)** | **2.02** | **2.67** | **2.73** | **2.19** |

**Table S3.** Electrochemical performance comparison between CWO-M and other vacancy materials in the literature.

| **Materials** | **Capacity at 1C (mAh g^-1^)** | | **Capacity at 2C (mAh g^-1^)** | | **Capacity retention** | **Reference** |
| --- | --- | --- | --- | --- | --- | --- |
| CWO-M | | 909 | | 768 | 91.1%/100 cycles at 0.2C | This work |
| CC/Co@CoO_1-x_ | | 897 | | 701 | 85.2%/100 cycles at 0.5C | [11] |
| CNT-LDH/Ar | | 845.1 | | 730.1 | 91.2%/100 cycles at 0.2C | [12] |
| STMn_0.3_ | | 767 | | 682 | 67.3%/500 cycles at 1C | [13] |
| Co@TiO_2-x_ | | 878 | | 777 | 82%/100 cycles at 0.2C | [14] |
| p-Co_3_O_4_/n-TiO_2_-HPs | | 925 | | 784 | 79.5%/100 cycles at 0.2C | [15] |
| WO@NC | | 724 | | 716 | 84.7%/100 cycles at 0.2C | [16] |
| OV–T_n_QDs@PCN | | 718 | | 672 | 90%/100 cycles at 0.1C | [17] |
| Cu_0.1_Co_0.9_P/MXene | | 872 | | 763 | 73.4%/100 cycles at 0.2C | [18] |
| CNT@TiO_2-_*_x_* | | 717 | | 597 | 66.9%/100 cycles at 0.2C | [19] |
| DHCP | | 800 | | 706 | 86.2%/200 cycles at 0.5C | [20] |

**Reference**

[1] G. Kresse, J. Hafner, *J.* *Phys. Rev. B* **1993**, 48, 13115.

[2] G. Kresse, J. Hafner, *J. Phys. Rev. B* **1994**, 49, 14251.

[3] G. Kresse, J. Furthmüller, *J. Comput. Mater. Sci.* **1996**, 6, 15.

[4] G. Kresse, J. Furthmüller, *J. Phys. Rev. B* **1996**, 54, 11169.

[5] J. P. Perdew, K. Burke, M. Ernzerhof, *Phys. Rev. Lett.* **1996**, 77, 3865.

[6] P. E. Blöchl, *J. Phys. Rev. B* **1994**, 50, 17953.

[7] S. Grimme, J. Antony, S. Ehrlich, H. Krieg, *J. Chem. Phys.* **2010**, 132.

[8] M. J. Piotrowski, C. G. Ungureanu, P. Tereshchuk, K. E. Batista, A. S. Chaves, D. Guedes-Sobrinho, J. L. Da Silva, *J. Phys. Chem. C* **2016**, 120, 28844.

[9] V. Wang, N. Xu, J. C. Liu, G. Tang, W. T. Geng, *Comput. Phys. Commun.* **2021**, 267, 108033.

[10] J. K. Nørskov, J. Rossmeisl, A. Logadottir, L. Lindqvist, J. R. Kitchin, T. Bligaard, H. Jonsson, *J. Phys. Chem. B* **2004**, 108, 17886.

[11] D. Fang, G. Wang, S. Huang, T. Chen Li, J. Yu, D. Xiong, D. Yan, X. Liang Li, J. Zhang, Y. Von Lim, S. A. Yang, H. Ying Yang, *Chem. Eng. J.* **2021**, 411, 128546.

[12] C. Li, Y. Zhao, Y. Zhang, D. Luo, J. Liu, T. Wang, W. Gao, H. Li, X. Wang, *Chem. Eng. J.* **2021**, 417, 129248.

[13] W. Hou, P. Feng, X. Guo, Z. Wang, Z. Bai, Y. Bai, G. Wang, K. Sun, *Adv. Mater.* **2022,** 34, 2202222.

[14] Y. Li, X. Zhang, G. Liu, A. Gerhardt, K. Evans, A. Jia, Z. Zhang, *J. Energy Chem.* **2020**, 48, 390.

[15] H. Li, C. Chen, Y. Yan, T. Yan, C. Cheng, D. Sun, L. Zhang, *Adv. Mater.* **2021**, 33, 2105067.

[16] S. Wang, Y. Wang, Y. Song, X. Jia, J. Yang, Y. Li, J. Liao, H. Song, *Energy Storage Mater.* **2021**, 43, 422.

[17] H. Zhang, L. Yang, P. Zhang, C. Lu, D. Sha, B. Yan, W. He, M. Zhou, W. Zhang, L. Pan, Z. Sun, *Adv. Mater.* **2021**, 33, 2008447.

[18] J. Shan, W. Wang, B. Zhang, X. Wang, W. Zhou, L. Yue, Y. Li, *Adv. Sci.* **2022**, 9, 2204192.

[19] Y. Wang, R. Zhang, J. Chen, H. Wu, S. Lu, K. Wang, H. Li, C. J. Harris, K. Xi, R. V. Kumar, S. Ding, *Adv. Energy Mater.* **2019**, 9, 1900953.

[20] P. Zeng, X. Zhou, J. Peng, X. Huang, B. Chang, G. Chen, M. Chen, L. Zheng, Y. Pei, J. Su, X. Wang, *Adv. Funct. Mater.* **2023**, 33, 2211818.

1. * **Corresponding author:** Tel.: +86 73158292060; fax: +86 73158292061.

   **Email:** hongboshu@xtu.edu.cn (H. Shu), xingqiaowu@wzu.edu.cn (X. Wu), mfchen@xtu.edu.cn (M. Chen).

   #These authors contributed equally to this work. [↑](#footnote-ref-1)
